# Sex-specific infarct volume associations and early prediction of language impairment progression following stroke surgery: a network approach

**DOI:** 10.3389/fneur.2025.1728433

**Published:** 2025-12-03

**Authors:** Guo-Dong Li, Zhong-Hai Tao, Ye-Kai Chen, Zhou-Kang Wu, Zi-Ning Zhu, Bai-Hui Luo, Yu-Han Chen, Shan-Shan Ma, Bing Fu, Hui Zheng

**Affiliations:** 1 Department of Neurology, Lianyungang Second People’s Hospital Affiliated to Kangda College of Nanjing Medical University, Lianyungang, Jiangsu, China; 2 School of Psychology, Zhejiang Normal University, Jinhua, China; 3 Department of Psychology, School of Humanities and Social Sciences, University of Science and Technology of China, Hefei, China; 4 Department of Philosophy, Zhenjiang Mental Health Center, Zhenjiang, Jiangsu, China; 5 School of Physical Education and Health, Guangzhou City Construction College, Guangzhou, Guangdong, China; 6 Kangda College of Nanjing Medical University, Lianyungang, Jiangsu, China; 7 Shanghai Key Laboratory of Psychotic Disorders, Brain Health Institute, National Centre for Mental Disorders, Shanghai Mental Health Centre, Shanghai Jiao Tong University School of Medicine, Shanghai, China

**Keywords:** stroke, language impairment progression, neurological deterioration, predictive model, LASSO regression, network analysis, Glasgow Coma Scale, sex-specific associations

## Abstract

**Background:**

Post-stroke language impairment affects nearly one-third of acute stroke patients or 30% of ischemic stroke patients, yet predicting its progression remains challenging due to multifactorial recovery processes.

**Methods:**

Predictors were selected using Least Absolute Shrinkage and Selection Operator (LASSO) regression. The interrelationships among these predictors and their mechanistic links to gender and post-stroke language impairment were subsequently explored employing Graphical LASSO (GLASSO) and Bayesian network analysis. Furthermore, Network Outcome Analysis (NOA) was applied to investigate the associations between preoperative predictors and the severity of postoperative language impairment deterioration.

**Results:**

LASSO identified eight predictors for inclusion in the model. The ROC analysis demonstrated favorable predictive efficacy (AUC = 0.80 [95% CI: 0.719–0.876], accuracy = 0.73, sensitivity = 0.78, specificity = 0.69, PPV = 0.71, NPV = 0.76). The DCA results (probability range: 0–0.81, 0.91) further indicated good clinical utility of the model. Preoperative GCS emerged as the primary direct predictor. Although male patients exhibited larger infarct volumes (22.72 mL vs. 15.19 mL in females), this difference was not directly associated with poorer language outcomes.

**Discussion:**

This multimodal model, enhanced by network analysis, accurately predicts language impairment progression and highlights preoperative consciousness as a key mediator, supporting precision stroke rehabilitation by capturing complex predictor interrelationships.

## 1 Introduction

Stroke remains a leading cause of long-term disability worldwide, with neurological deterioration during the acute phase significantly impacting recovery outcomes and rehabilitation strategies (1). Among the various post-stroke impairments, language impairment—a motor speech disorder affecting articulation—occurs in approximately one-third of acute stroke patients or 30% of ischemic stroke patients, and serves as a sensitive marker of neurological deterioration (2, 3). Early prediction of language impairment worsening is clinically crucial, as timely identification allows for targeted speech therapy interventions and improved communication outcomes (2). However, predicting which patients will experience language impairment progression remains challenging due to the multifactorial nature of stroke recovery.

Traditional stroke severity assessments, including the National Institutes of Health Stroke Scale (NIHSS), Glasgow Coma Scale (GCS), and modified Rankin Scale (mRS), provide valuable baseline measures but demonstrate limited accuracy in predicting specific neurological deterioration patterns (4, 5). While these clinical scales capture acute stroke severity, they fail to account for critical imaging features such as infarct volume and location, which are known to influence speech outcomes (6, 7). Furthermore, most existing prediction models employ univariate approaches that overlook complex interactions among clinical, biochemical, and imaging factors (8). For instance, studies have shown that infarct volume impacts recovery differently across sexes, with males exhibiting stronger associations between lesion size and functional outcomes (9, 10). The lack of models that simultaneously consider these multimodal factors and their interrelationships represents a significant gap in stroke prognostication.

Recent advances in machine learning and network analysis offer promising solutions to address these limitations. Least Absolute Shrinkage and Selection Operator (LASSO) regression enables efficient identification of the most predictive variables from high-dimensional datasets, reducing model complexity while maintaining accuracy (11). Beyond variable selection, network analysis methods including Graphical LASSO (GLASSO) and Bayesian networks can map complex interdependencies among predictors, revealing non-obvious relationships that traditional regression models miss (12). For example, here we use GLASSO to identify whether infarct volume directly influences language impairment deterioration or operates through indirect pathways via consciousness level or functional independence. Similarly, Bayesian networks can establish directional relationships, distinguishing causal predictors from mere correlates (13). Despite their potential, these advanced analytical techniques remain underutilized in stroke recovery research, particularly for predicting specific neurological outcomes like language impairment worsening.

Here we report on the development and validation of a comprehensive predictive model for early language impairment progression following stroke surgery by integrating clinical assessments (NIHSS, GCS, mRS, Activities of Daily Living scale [ADL], Clinical Dementia Rating [CDR], Alberta Stroke Program Early CT Score [ASPECTS], Hachinski Ischemic Scale [HIS]), biochemical markers, and CT-derived infarct volume and affected brain regions. We employ LASSO regression to identify core predictors from this multimodal dataset, then apply GLASSO and Bayesian network analyses to elucidate the mechanistic pathways linking predictors to language impairment outcomes. A particular focus is placed on investigating sex- specific associations with infarct volume, as these relationships may inform personalized rehabilitation strategies. We hypothesize that: (1) a multimodal model incorporating clinical scales, biomarkers, and imaging features will achieve superior predictive accuracy for language impairment deterioration compared to single-domain approaches; (2) network analysis will reveal that infarct volume influences language impairment worsening through indirect pathways mediated by consciousness level and functional status, with sex-specific effects; and (3) preoperative assessments will demonstrate stronger predictive value than postoperative measures, enabling earlier clinical decision-making.

## 2 Methods

### 2.1 Study design

This study employed a cross-sectional cohort design. The data for this study were obtained from stroke patients who have undergone emergency cerebrovascular intervention surgery in a hospital in China and encompassed multiple data modalities, including biochemical indicators, cerebral infarct volume, CT imaging, and standardized questionnaire assessments. Data collection spanned 4 years, from January 2022 to September 2025, with a total of 204 patients enrolled. Data were considered valid only when all 34 specified indicators were completely recorded or assessed. A total of 164 patients were included in the analysis. In observational studies, it is generally recommended that the sample size be 10 to 20 times the number of dependent variables. We plan to include the top 10 variables with nonzero coefficients (or all variables if fewer than 10) as predictors. In the most extreme case, with 10 predictors, one demographic variable (gender), and one dependent variable, the minimum required sample size would be 120. Our study sample size meets and exceeds this minimum requirement. CT imaging assessments and certain questionnaires were designed with a pretest-posttest design. Language impairment was assessed using the ninth item (Language) of the NIHSS questionnaire. Patients underwent preoperative assessment within 2 h prior to emergency cerebrovascular intervention surgery, followed by a postoperative assessment on the second day after surgery (approximately 48 h post-intervention). The difference between the postoperative and preoperative assessments was used to evaluate the degree of deterioration in stroke-related neurological function. All personnel involved in the project received professional training to ensure the validity and consistency of data collection. This study was approved by the Institutional Review Board (IRB) of the Second People’s Hospital of Lianyungang (Approval No. 2025K096). The study was conducted in accordance with local regulations and institutional requirements.

The data analysis process consisted of six steps: 1. Comparing of differential indicators between male and female groups. 2. Comparing the differential indicators between groups with different degrees of language impairment progression. 3. Using LASSO to select core predictive factors for language impairment progression and testing them with ROC and DCA, thereby establishing a reliable predictive model for this speech disorder. 4. Using GLASSO network analysis to explore the influencing mechanisms among core predictive factors, gender, and language impairment progression. 5. Building upon the previous step, using Bayesian network analysis to test the directionality of different influencing paths. 6. Exploring the impact of preoperative predictive factors on postoperative language impairment progression through network outcome analysis (NOA).

### 2.2 Measurement

The NIHSS is used to assess the degree of neurological deterioration in stroke patients (14). The NIHSS comprises 11 subdomains, including level of consciousness, gaze, visual fields, facial palsy, motor function of the upper limb, motor function of the lower limb, limb ataxia, sensory function, language, dysarthria, and neglect. Post-stroke language impairment progression (measured as the deterioration score, LANG) is defined here as the difference between the preoperative and postoperative NIHSS subdomain scores for language (postoperative score minus preoperative score). A LANG value of less than zero was classified as improvement; all other values constituted the non-improvement group.

The factors influencing the progression of post-stroke language impairment.

The patients’ post-stroke self-care ability, level of consciousness, cognitive function, extent of ischemic injury, overall degree of functional impairment, and vascular dementia were evaluated using six assessment tools: ADL (15), GCS (16), CDR (17), ASPECTS (18), mRS (19), and HIS (20). In addition, biochemical data were obtained from routine clinical blood draws upon admission. Cerebral infarct volume was quantified from admission non-contrast CT scans using a validated semi-automated hemispheric-asymmetry workflow implemented in the FMRIB Software Library (FSL). Each scan was first reoriented to the standard neurological convention (fslreorient2std), skull-stripped with a CT-optimized fractional intensity threshold, resampled to 1.0 mm isotropic resolution, and intensity-normalized within a Hounsfield Unit window of −20 to 80. A left–right flipped image was rigidly registered back to the original image using FLIRT (21), and an absolute voxel wise difference map was computed to highlight hemispheric asymmetry. The map was thresholded at the 98th percentile of its intensity distribution, and clusters smaller than 500 mm^3^ were removed, yielding the final lesion mask. The mask was rigidly aligned to the MNI152 1 mm template for spatial standardization, and infarct volumes were expressed in mL. All lesion masks underwent visual quality control by experienced raters, and the full methodological details are provided in the Supplementary materials.

In this study, the predictive factors were coded using standardized abbreviations for clarity. Functional status at admission was represented by PQ. Postoperative imaging findings were assessed using PSQ3 (ASPECTS), while discharge outcomes were captured by PSQ6 (mRS). Biochemical marker was represented by BIOC. In addition, VOL (infarct volume), CE (damaged brain regions), and LANG (language impairment score) were also incorporated into the analysis.

### 2.3 Quality control

The original data were obtained from clinical practice, with a total of 204 stroke patients enrolled. As certain predictive factors are measured infrequently in stroke patients, simply excluding missing values would lead to a significant reduction in sample size. To ensure an adequate sample size, factors with excessive missing values were excluded, specifically the postoperative 3-month and 12-month mRS follow-up scores, as well as homocysteine levels. Subsequently, samples with missing data were removed, resulting in a final sample size of 164 valid cases. Finally, the completeness of the data was verified through visualization of missing data, providing a solid foundation for subsequent analysis.

### 2.4 Establishment of the predictive model

To identify the key variables influencing language impairment progression, the LASSO algorithm was employed for variable selection. LASSO is a machine learning method that applies L1 regularization to shrink some regression coefficients to zero, thereby eliminating unimportant or redundant variables and facilitating the identification of core features with predictive value.

In this study, biochemical indicators (BIOC), infarct volume (VOL), cerebral affected region (CE), preoperative questionnaire scores (PQ), and postoperative questionnaire scores (PSQ) were included as predictive variables, while the LANG was defined as the dependent variable. Variables with non-zero coefficients after LASSO selection were identified as the key influencing factors associated with language impairment progression.

To evaluate the performance of the model, Receiver Operating Characteristic (ROC) analysis and Decision Curve Analysis (DCA) were conducted. In these analyses, LANG values less than 0 were coded as 1, and all others as 0. ROC analysis was used to assess the predictive accuracy of the model, with the area under the curve (AUC) serving as a key metric. An AUC value closer to 1 indicates superior model performance, with the 95% confidence interval calculated using the DeLong method. DCA was used to evaluate the clinical utility and net benefit of the predictive model, employing 2000 bootstrap resampling iterations to assess net benefit, with threshold values ranging from 0 to 1 to cover clinical decision-making scenarios. The model’s curve achievement of a higher net benefit than the “treat-all” and “treat-none” strategies across a broader range of threshold values indicates greater clinical applicability and utility of the model.

### 2.5 Network analysis

GLASSO network analysis was applied to construct a regularized network structure to evaluate the strength and centrality of associations among the selected core variables, gender, and language impairment progression following stroke by quickNet R package^1^. This method effectively reveals the interrelationships among core predictive factors, gender, and language impairment progression outcomes. The centrality measures in the network analysis include Strength, Closeness, Betweenness, and Expected Influence. Strength represents the total intensity of connections for a given node, Closeness measures the average distance from a node to all other nodes, Betweenness assesses the bridging role of a node, and Expected Influence is used to assess the potential impact of a node on other nodes. In the visualization of the network analysis, due to the limited sample size obtained from clinical practice, no fixed threshold was set to better represent the relationships among core predictive factors, gender, and language impairment progression. This approach allowed for a more flexible representation of the interconnections and their potential impacts.

Bayesian network analysis using the bnlearn R package was employed to explore the causal relationships among the core predictive factors, gender, and changes in LANG (22). The hc algorithm was utilized to infer the underlying network structure. The obtained structure was subsequently adapted to the data domain, with arc weights estimated via 50,000 bootstrap resampling iterations. The constructed network was visualized using the qgraph R package to illustrate the relationships among variables. The direction of each edge was determined based on the predominant direction observed during resampling. During the resampling process, if at least 51% of the bootstrapped networks indicated a directed edge from node A to node B, this directionality was represented in the final visualization by an arrow pointing from A to B.

Subsequently, based on the above findings, NOA was further conducted using the quickNet R package. NOA integrates baseline predictive variables and follow-up outcome measures within a single network, allowing for a more comprehensive evaluation of the influence of predictive factors on outcome variables. The predictive variables included those selected from the preoperative assessment indicators and incorporated into the final predictive model, while the outcome variable represented the degree of language impairment progression following stroke. The parameter settings and centrality measures for the NOA network analysis were the same as those used in the GLASSO network.

### 2.6 Statistical analyses

All statistical analyses were performed using R. Except for the biochemical indicators of Cl and infarct volume, the remaining variables were non-normally distributed. Categorical data were presented as *n* (%), and between-group comparisons were performed using the chi-square test or Fisher’s exact test (when the expected frequency was less than 5), with effect sizes evaluated by Cramer’s V. Continuous data are expressed as mean ± standard deviation, with between-group comparisons analyzed by independent samples t-tests and effect sizes quantified using Cohen’s d. The *p*-values were adjusted for multiple comparisons using the Benjamini-Hochberg method. LASSO regression was primarily conducted using the glmnet R package, the ROC curve was plotted with pROC and ggplot2 packages, and the DCA curve was drawn using the rmda package. Network analysis was primarily performed using the quickNet integrated package, the bnlearn package, and the qgraph package for computation or visualization. The alpha level was set at 0.05.

## 3 Results

### 3.1 Comparison of parameters between patients of different sexes

As shown in Table 1, among the 164 included patients, 101 were male and 63 were female. Compared to females, on average males were younger (69.39 ± 10.72 vs. 73.51 ± 9.38; *t* = 2.59, *p* < 0.05, Cohen’s d = 0.40), had lower lactate dehydrogenase levels (208.32 ± 48.95 vs. 234.25 ± 71.64; *t* = 2.53, *p* < 0.05, Cohen’s d = 0.44), and had larger infarct volumes (22.72 ± 6.65 vs. 15.19 ± 6.36; *t* = −7.24, *p* < 0.001, Cohen’s d = −1.15). The indicator that remained significant after Benjamini-Hochberg correction was infarct volume, with an adjusted *p*-value of <0.001. No significant differences were observed for other indicators.

**Table 1:** Comparison of parameters among language impairment patients by sex.

Category	Male (*n* = 101)	Female (*n* = 63)	*t*/*χ*^2^ value	*p* value	Adjusted *p* value	Effect size
Age (years)	69.39 ± 10.72	73.51 ± 9.38	2.59	0.011	0.148	0.40
LANG	−0.39 ± 0.77	−0.21 ± 0.54	1.74	0.083	0.471	0.26
Admission						
ADL (Score)	20.69 ± 15.46	20.24 ± 15.69	−0.18	0.856	0.989	−0.03
GCS (Score)	8.90 ± 2.22	8.54 ± 2.58	−0.92	0.360	0.816	−0.15
CDR (Score)	8.88 ± 4.80	9.37 ± 4.86	0.62	0.534	0.925	0.10
ASPECT (Score)	9.58 ± 0.78	9.60 ± 0.71	0.16	0.872	0.989	0.03
mRS (Score)	4.35 ± 0.70	4.33 ± 0.74	−0.11	0.910	0.998	−0.02
HIS (Score)	10.60 ± 2.05	10.68 ± 2.35	0.22	0.828	0.989	0.04
Infarct Volume (mL)	22.72 ± 6.65	15.19 ± 6.36	−7.24	<0.001	<0.001	−1.15
The Affected Hemisphere (left/%)	43/42.6%	23/36.5%	0.37^a^	0.544	0.925	0.00^b^
Cerebellar Damage (Yes/%)	26/25.7%	19/30.2%	0.19^a^	0.662	0.989	0.00^b^
Language Area Damage (Yes/%)	7/6.9%	3/4.8%		0.743	0.989	0.02^b^
Broca’s area Damage (Yes/%)	2/2.0%	0/0%		0.524	0.925	0.03^b^
Wernicke’s area Damage (Yes/%)	5/5.0%	3/4.8%		1.000	1.000	0.00^b^
K (mmol/L)	4.01 ± 0.42	3.88 ± 0.48	−1.76	0.080	0.471	−0.29
Na (mmol/L)	137.08 ± 3.58	137.36 ± 3.61	0.49	0.622	0.989	0.08
Cl (mmol/L)	102.89 ± 3.33	102.48 ± 4.20	−0.66	0.511	0.925	−0.11
CO2 Binding Capacity (mmol/L)	26.01 ± 19.13	23.91 ± 2.94	−1.08	0.282	0.799	−0.14
Neutrophil (10^9)	7.18 ± 8.83	7.18 ± 4.08	0.00	0.996	1.000	0.00
Lymphocytes (10^9)	1.57 ± 1.61	1.35 ± 0.69	−1.19	0.234	0.728	−0.16
Monocytes (10^9)	0.55 ± 0.44	0.53 ± 0.47	−0.31	0.758	0.989	−0.05
C-reactive protein (mg/L)	11.83 ± 21.67	12.79 ± 19.94	0.29	0.773	0.989	0.05
INR Coagulation	1.13 ± 0.13	1.13 ± 0.12	−0.22	0.826	0.989	−0.03
D-Dimer (ng/mL)	610.68 ± 1184.42	436.51 ± 364.69	−1.38	0.171	0.646	−0.18
Fibrinogen (ng/mL)	64.63 ± 608.56	3.99 ± 0.91	−1.00	0.319	0.816	−0.13
Lactate Dehydrogenase (U/L)	208.32 ± 48.95	234.25 ± 71.64	2.53	0.013	0.148	0.44
Blood Glucose (mmol/L)	7.14 ± 2.78	8.06 ± 4.40	1.49	0.141	0.611	0.26
Postoperative						
ASPECT (Score)	7.11 ± 2.79	6.83 ± 2.69	−0.65	0.518	0.925	−0.10
Triglycerides (mmol/L)	1.36 ± 0.76	1.36 ± 0.78	−0.01	0.994	1.000	0.00
Total Cholesterol (mmol/L)	4.38 ± 1.21	4.14 ± 1.27	−1.19	0.235	0.728	−0.19
HDL (mmol/L)	1.14 ± 0.31	1.23 ± 0.32	1.90	0.059	0.471	0.31
LDL (mmol/L)	2.74 ± 0.83	2.61 ± 0.83	−0.95	0.345	0.816	−0.15
Glycated Hemoglobin (4–6%)	6.76 ± 1.69	6.68 ± 1.71	−0.27	0.785	0.989	−0.04
Discharge						
mRS (Score)	3.38 ± 1.14	3.67 ± 1.28	1.47	0.144	0.611	0.24

^a^Chi-square test. Independent two-sample *t*-test (two-tailed) was used to compare means between groups. Fisher’s exact test was applied to analyze the categorical variables of Language Area Damage, Broca’s Area Damage, and Wernicke’s Area Damage, as the assumption for the Chi-square test was violated (i.e., expected counts in these variables were < 5). Statistically significant differences are marked with a *p*-value less than 0.05. Effect sizes were assessed using ^b^Cramer’s V for chi-square / fisher’s exact tests and Cohen’s d for *t*-tests. LANG, the deterioration score of National Institutes of Health Stroke Scale; ADL, Activities of Daily Living; GCS, Glasgow Coma Scale; CDR, Clinical Dementia Rating; ASPECT, Alberta Stroke Program Early CT Score; mRS, modified Rankin Scale; HIS, Hachinski Ischemic Scale; K, Potassium; Na, Sodium; Cl, Chlorine; INR, International Normalized Ratio; HDL, High-density lipoprotein; LDL, Low-density lipoprotein. Some values appear as 0 due to their very small magnitude; however, for statistical measures that include directionality and require verification, please refer to Supplementary Results 1.

### 3.2 Comparison of parameters among patients with different degrees of language impairment progression

As shown in Table 2, this study included a total of 164 stroke patients who underwent surgery, among whom 45 patients exhibited postoperative improvement in language function (LANG < 0), while 119 patients showed no improvement (LANG ≥ 0). Compared to the non-improvement group, patients in the improvement group demonstrated significantly lower CDR scores at admission (7.41 ± 3.92 vs. 9.69 ± 4.98; *t* = 3.08, *p* = 0.003, Cohen’s d = 0.48), lower GCS scores at admission (7.82 ± 2.33 vs. 9.12 ± 2.29; *t* = 3.20, *p* = 0.002, Cohen’s d = 0.56), lower HIS scores at admission (10.09 ± 1.83 vs. 10.84 ± 2.25; *t* = 2.20, *p* < 0.05, Cohen’s d = 0.35), and lower mRS scores at discharge (3.02 ± 1.14 vs. 3.66 ± 1.18; *t* = 3.19, *p* = 0.002, Cohen’s d = 0.55). In addition, their Cl values were significantly lower (101.76 ± 3.91 vs. 103.10 ± 3.54; *t* = 2.02, *p* < 0.05, Cohen’s d = 0.37). Conversely, the improvement group demonstrated higher ASPECTS scores at postoperative (7.78 ± 1.91 vs. 6.71 ± 2.96; *t* = −2.73, *p* = 0.007, Cohen’s d = −0.40) and larger infarct volumes (22.07 ± 7.75 vs. 18.98 ± 7.23; *t* = −2.32, *p* < 0.05, Cohen’s d = −0.42). The three indicators that remained significant after Benjamini-Hochberg correction were the CDR admission score, GCS admission score, and mRS discharge score, with adjusted *p*-values all equal to 0.030. No significant differences were observed in the remaining parameters between the two groups.

**Table 2:** Table of comparison of parameters among patients with different degrees of deterioration in language impairment.

Category	Postoperative improvement (*n* = 45)	Postoperative no improvement (*n* = 119)	*t*/*χ*^2^ value	*p* value	Adjusted *p* value	Effect size
Gender (man/%)	32/71.1%	69/58.0%	1.86^a^	0.173	0.490	0.09^b^
Age (years)	69.19 ± 12.34	71.64 ± 9.53	1.21	0.233	0.494	0.24
Admission						
ADL (Score)	17.44 ± 11.66	21.68 ± 16.62	1.83	0.069	0.263	0.27
GCS (Score)	7.82 ± 2.33	9.12 ± 2.29	3.20	0.002	0.030	0.56
CDR (Score)	7.41 ± 3.92	9.69 ± 4.98	3.08	0.003	0.030	0.48
ASPECT (Score)	9.51 ± 0.87	9.62 ± 0.70	0.77	0.447	0.672	0.15
mRS (Score)	4.31 ± 0.60	4.35 ± 0.75	0.37	0.711	0.835	0.06
HIS (Score)	10.09 ± 1.83	10.84 ± 2.25	2.20	0.031	0.173	0.35
Infarct Volume (mL)	22.07 ± 7.75	18.98 ± 7.23	−2.32	0.023	0.156	−0.42
The Affected Hemisphere (left/%)	14/31.1%	52/43.7%	1.66^a^	0.198	0.494	0.08
Cerebellar Damage (Yes/%)	14/31.1%	31/26.1%	0.20^a^	0.651	0.820	0.00
Language Area Damage (Yes/%)	3/6.7%	7/5.9%		1.000	1.000	0.00
Broca’s area Damage (Yes/%)	1/2.2%	1/0.8%		0.475	0.672	0.00
Wernicke’s area Damage (Yes/%)	2/4.4%	6/5.0%		1.000	1.000	0.00
K (mmol/L)	4.00 ± 0.41	3.95 ± 0.46	−0.73	0.468	0.672	−0.12
Na (mmol/L)	136.32 ± 4.74	137.52 ± 2.99	1.58	0.119	0.405	0.34
Cl (mmol/L)	101.76 ± 3.91	103.10 ± 3.54	2.02	0.047	0.217	0.37
CO2 Binding Capacity (mmol/L)	24.20 ± 2.96	25.58 ± 17.67	0.83	0.411	0.672	0.09
Neutrophil (10^9)	7.12 ± 3.88	7.20 ± 8.32	0.08	0.938	1.000	0.01
Lymphocytes (10^9)	1.33 ± 0.74	1.55 ± 1.50	1.24	0.216	0.494	0.16
Monocytes (10^9)	0.48 ± 0.18	0.56 ± 0.52	1.52	0.132	0.407	0.18
C-reactive protein (mg/L)	13.83 ± 23.79	11.58 ± 19.87	−0.56	0.576	0.753	−0.11
INR Coagulation	1.13 ± 0.12	1.13 ± 0.13	−0.16	0.870	0.986	−0.03
D-Dimer (ng/mL)	542.31 ± 508.19	544.33 ± 1082.78	0.02	0.987	1.000	0.00
Fibrinogen (ng/mL)	4.19 ± 0.89	55.39 ± 560.65	1.00	0.321	0.607	0.11
Lactate Dehydrogenase (U/L)	223.27 ± 55.37	216.39 ± 61.58	−0.69	0.494	0.672	−0.11
Blood Glucose (mmol/L)	8.08 ± 4.54	7.27 ± 3.01	−1.11	0.273	0.547	−0.23
Postoperative						
ASPECT (Score)	7.78 ± 1.91	6.71 ± 2.96	−2.73	0.007	0.062	−0.40
Triglycerides (mmol/L)	1.44 ± 0.86	1.33 ± 0.73	−0.74	0.459	0.672	−0.14
Total Cholesterol (mmol/L)	4.49 ± 1.39	4.21 ± 1.17	−1.22	0.227	0.494	−0.23
HDL (mmol/L)	1.19 ± 0.33	1.17 ± 0.31	−0.37	0.713	0.835	−0.07
LDL (mmol/L)	2.79 ± 0.92	2.65 ± 0.80	−0.86	0.393	0.672	−0.16
Glycated Hemoglobin (4–6%)	7.21 ± 2.05	6.55 ± 1.50	−1.99	0.051	0.217	−0.40
Discharge						
mRS (Score)	3.02 ± 1.14	3.66 ± 1.18	3.19	0.002	0.030	0.55

^a^ Chi-square test. Independent two-sample t-test (two-tailed) was used to compare means between groups. Fisher’s exact test was applied to analyze the categorical variables of Language Area Damage, Broca’s Area Damage, and Wernicke’s Area Damage, as the assumption for the Chi-square test was violated (i.e., expected counts in these variables were < 5). Statistically significant differences are marked with a *p*-value less than 0.05. Effect sizes were assessed using ^b^ Cramer’s V for chi-square / fisher’s exact tests and Cohen’s d for *t*-tests. ADL, Activities of Daily Living; GCS, Glasgow Coma Scale; CDR, Clinical Dementia Rating; ASPECT, Alberta Stroke Program Early CT Score; mRS, modified Rankin Scale; HIS, Hachinski Ischemic Scale; K, Potassium; Na, Sodium; Cl, Chlorine; INR, International Normalized Ratio; HDL, High-density lipoprotein; LDL, Low-density lipoprotein. Some values appear as 0 due to their very small magnitude; however, for statistical measures that include directionality and require verification, please refer to Supplementary Results 1.

### 3.3 Predictive model

The results of the LASSO analysis revealed eight core predictive variables, including VOL (infarct volume), CE1 (the affected hemisphere), PQ3 (ADL score at admission), PQ5 (GCS score at admission), BIOC4 (C-reactive protein 0–10 mg/L), BIOC19 (glycated hemoglobin 4–6%), PSQ3 (ASPECTS score after surgery), and PSQ6 (mRS score at discharge). These eight variables were subsequently incorporated into the model as core predictors.

**Figure 1:**
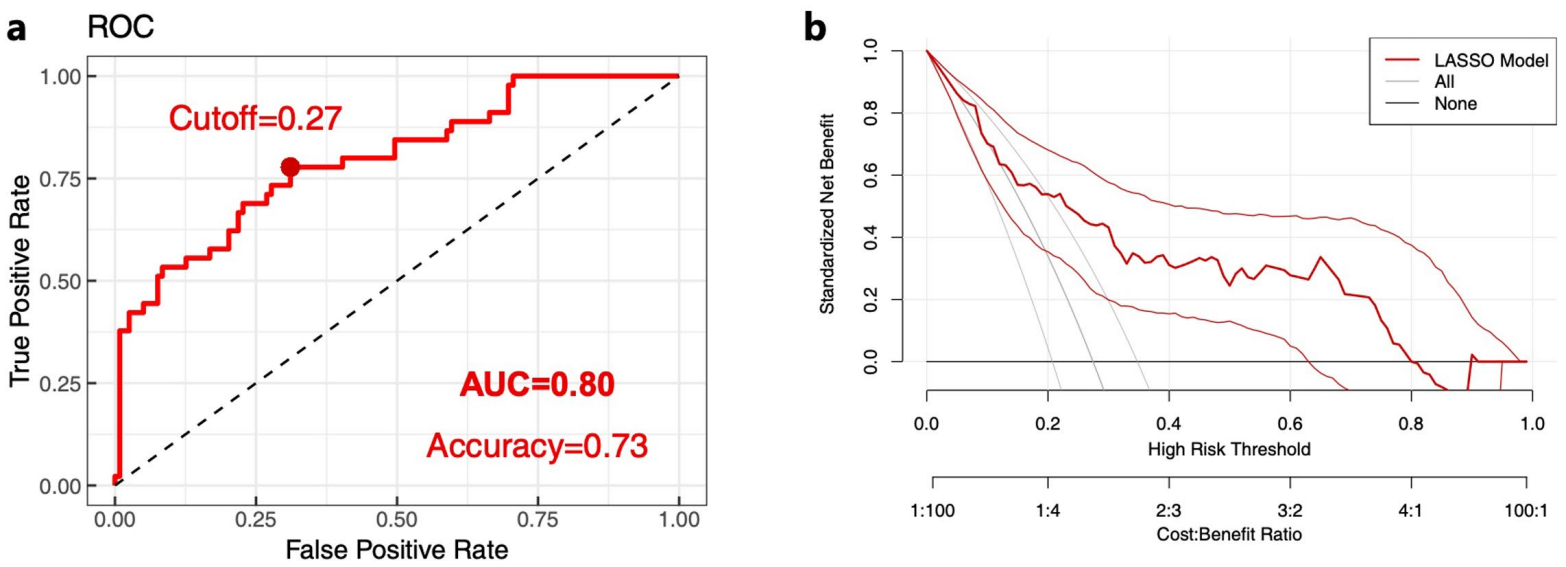
Validation of the language impairment progression prediction model based on LASSO regression. **(a)** Core predictive factors ROC curve validation plot. **(b)** Core predictive factors DCA validation plot. The closer the AUC and accuracy values are to 1, the better the predictive performance of the model. In the DCA, the LASSO model demonstrates a wider threshold range above the None and All strategy curves, indicating greater clinical applicability of the model.

Subsequently, the predictive performance of the LANG model was evaluated, demonstrating satisfactory predictive accuracy. The ROC curve analysis yielded an AUC of 0.80 (95% CI: 0.719–0.876), with an accuracy of 73.3%, sensitivity of 77.8%, specificity of 68.9%, positive predictive value (PPV) of 71.4%, and negative predictive value (NPV) of 75.6%, as shown in Figure 1a. The DCA result demonstrated that the model provided superior net benefit compared to the “None” and “All” strategies across a wide threshold probability range of 0–0.81, 0.91, as shown in Figure 1b.

### 3.4 Network results

**Figure 2:**
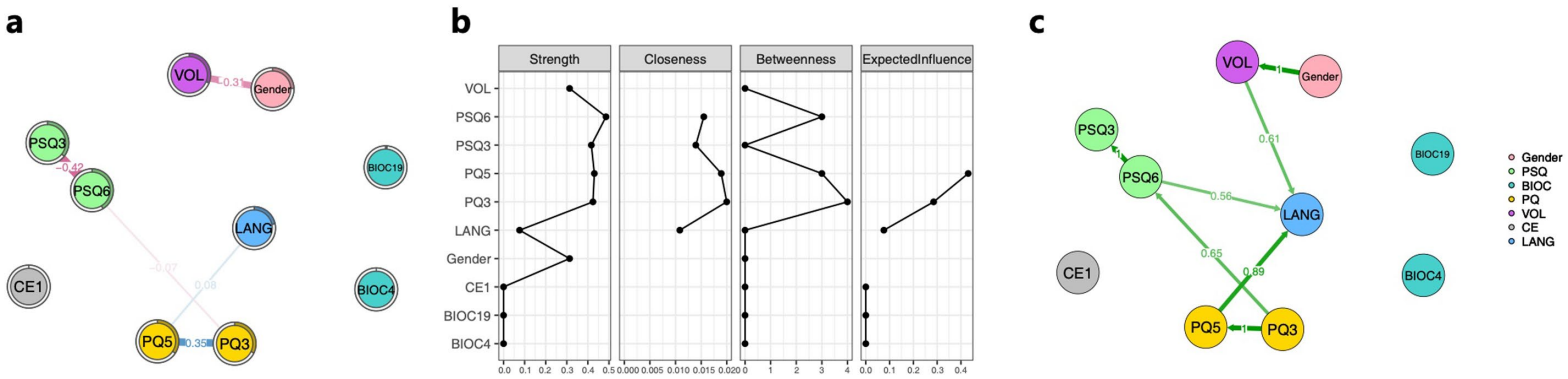
Association among core predictive variables, gender, and progression of language impairment. **(a)** GLASSO network composed of core predictive factors, gender, and the language impairment progression. **(b)** Centrality property of core predictive factors, gender, and the language impairment progression network. **(c)** Bayesian network consisting of core predictive factors, gender, and the language impairment progression. The nodes in the figure represent the filtered core predictive factors, gender, and the language impairment progression. The numerical values along the paths indicate the strength of associations or dependencies between connected variables. Blue lines denote positive correlations, while red lines indicate negative correlations; the thickness and boldness of the lines reflect the magnitude of these associations. The direction of the arrows represents the direction of influence between variables. Green lines in the figure illustrate the conditional dependencies among core predictive factors, gender, and language impairment worsening in the Bayesian network. PSQ3, ASPECTS postoperative score; PSQ6, mRS discharge score; BIOC4, C-reactive protein (0–10 mg/L); BIOC19, glycosylated hemoglobin (4–6%); PQ3, ADL admission score; PQ5, GCS admission score; VOL, cerebral infarction volume; CE1, the affected hemisphere; and LANG, degree of language impairment worsening.

The result of the GLASSO network analysis is shown in Figure 2a. PQ5 (preoperative GCS score) has a direct positive edge with LANG (weight = 0.08), while PQ3 (preoperative ADL score), PSQ3 (postoperative ASPECTS score), and PSQ6 (discharge mRS score) indirectly influence LANG through PQ5. Notably, VOL (infarct volume) shows a negative association with gender, with a weight of −0.31. The centrality indicators of the network analysis show that gender, PQ3, PQ5, PSQ3, PSQ6, and VOL play relatively central roles in the network, with Strength values greater than 0.3. Among them, PQ3, PQ5, and PSQ6 serve as strong bridges, with bridging occurrences of 8, 6, and 6, respectively (one unit in the figure represents two times). In the Expected Influence analysis, only PQ3 and PQ5 have positive values, specifically 0.29 and 0.43 respectively, as shown in Figure 2b.

The results of the Bayesian network analysis showed that VOL, PQ5, and PSQ6 had causal effects on LANG, with effect strengths of 0.61, 0.89, and 0.56, respectively. Consistent with the GLASSO results, a significant causal relationship was found between gender and VOL, with an effect strength of 1, as shown in Figure 2c.

**Figure 3:**
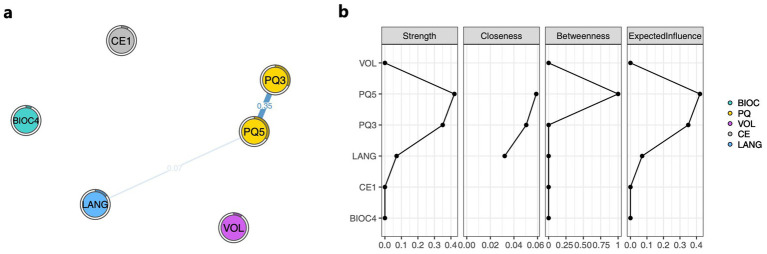
Network outcome analysis (NOA). **(a)** NOA composed of core predictive factors for language impairment progression prediction. **(b)** Centrality property of the NOA network analysis for language impairment progression prediction. The nodes in the figure consist of LANG and the core predictive factors measured at different time points from LANG. Blue lines indicate positive correlations, red lines represent negative correlations, and the thickness and boldness of the lines reflect the strength of the associations. Variables include BIOC4, C-reactive protein (0–10 mg/L); PQ3, ADL admission score; PQ5, GCS admission score; VOL, cerebral infarction volume; CE1, the affected hemisphere; and LANG, degree of language impairment worsening.

As shown in Figure 3a, in the NOA analysis, PQ5 exhibited a positive correlation with LANG, with a weight of 0.07, while PQ3 showed an indirect association with LANG. These results are consistent with the GLASSO network analysis, where PQ3 and PQ5 once again demonstrated their important roles. The centrality indicator results from the NOA network analysis revealed that PQ3 and PQ5 play the most central roles, with Strength values of 0.35 and 0.42, respectively, as shown in Figure 3b.

## 4 Discussion

This study developed a multimodal predictive model for language impairment progression following stroke surgery, achieving an AUC of 0.80 (95% CI: 0.719–0.876) with balanced clinical utility across threshold probabilities. LASSO regression identified eight core predictors from clinical, biochemical, and imaging domains, with preoperative consciousness level (GCS) and functional independence (ADL) demonstrating the strongest predictive value. Network analyses revealed two key mechanistic insights: first, GCS functions as a central hub through which other predictors influence language impairment outcomes, rather than operating as isolated risk factors; second, infarct volume exhibits a strong sex-specific association (weight = −0.31; Bayesian effect strength = 1.0), with male patients showing systematically larger lesions. These findings confirm our hypotheses that multimodal integration enhances prediction accuracy and that network analysis elucidates complex interrelationships overlooked by traditional univariate approaches.

The central role of preoperative GCS in both GLASSO and NOA networks—serving as the sole direct predictor of language impairment progression (edge weight = 0.08, 0.07) while bridging connections among ADL, ASPECTS, and mRS—revealed a critical mechanistic pathway. This finding is biologically plausible because the consciousness level reflects the integrity of ascending reticular activating systems and distributed cortical networks that also govern motor speech control (23, 24). The Bayesian network’s directional confirmation (GCS → language impairment, effect strength = 0.89) supports a causal interpretation, consistent with clinical observations that impaired arousal compromises compensatory mechanisms for speech production (25). Importantly, this hub architecture suggests that interventions targeting consciousness enhancement—such as minimizing sedation, optimizing sleep–wake cycles, or augmenting arousal pharmacologically—may yield cascading benefits including improved speech outcomes (26). The positive association (higher GCS predicts greater worsening) underscores the prognostic value of bedside consciousness assessment, enabling rapid risk stratification before surgery using readily available clinical tools rather than advanced imaging or biomarkers.

The strong negative association between infarct volume and male sex represents a novel contribution with implications for personalized prognosis. Our differential analysis demonstrated that males on average harbor significantly larger infarcts (mean 22.72 vs. 15.19 mL), yet this volume difference did not translate into proportionally worse language impairment outcomes, as evidenced by the absence of direct sex → language impairment edges in either network model. This dissociation aligns with recent evidence showing sex-specific lesion-outcome relationships, wherein males exhibit stronger volume-outcome associations but females demonstrate greater vulnerability to smaller yet still functionally consequential lesions in certain regions (27). Several mechanisms may explain this pattern: anatomical factors (larger brain volumes and cerebrovascular reserve in males), hormonal neuroprotection in premenopausal women limiting infarct expansion, and sex differences in stroke etiology producing distinct lesion characteristics (10). Clinically, these findings argue for sex-stratified prognostic thresholds and rehabilitation protocols, as traditional volume-based predictions may systematically underestimate risk in female patients with smaller but equally disabling lesions.

This study has several limitations that should be addressed in future research. Although left-hemisphere lesions were included as a core predictor in the LASSO model, no significant association with language impairment scores was observed in the chi-square or network analysis. This may be due to the small sample size in this study, which could have introduced bias, limited statistical power to detect weaker network connections, and precluded comprehensive subgroup analyses. Additionally, the single-center Chinese cohort may limit the generalizability of the findings to populations with different stroke etiologies or healthcare contexts. The cross-sectional design with short-term follow-up (2 days postoperative) cannot assess long-term language impairment trajectories or validate whether early deterioration predicts persistent speech deficits. Network stability could be improved through fixed thresholding with larger samples, and the reliance on the NIHSS language impairment subscale—a coarse 3-point measure—may have reduced sensitivity to subtle speech changes compared to detailed acoustic analyses. Unmeasured confounders including lesion location (e.g., involvement of precentral gyrus vs. subcortical structures), white matter hyperintensity burden, and premorbid cognitive reserve may partly explain observed associations. Future research should prioritize external validation in diverse cohorts, incorporation of advanced neuroimaging (DTI for white matter tract quantification, resting-state fMRI for network connectivity), longitudinal follow-up through rehabilitation phases, and sex-stratified analyses to determine whether distinct predictive models are clinically warranted. Finally, the short-term follow-up (48 h after surgery) captures acute language deterioration but cannot assess long-term aphasia trajectories. Future research should incorporate extended follow-up periods (3, 6, and 12 months) to validate whether early deterioration serves as a predictor of chronic language impairment.

## 5 Conclusion

In conclusion, this study developed a multimodal predictive model for post-stroke language impairment progression, achieving robust performance (AUC 0.80, 95% CI 0.719–0.876) with broad clinical decision thresholds. LASSO identified eight key predictors, with preoperative GCS and ADL exerting the strongest influence. Network analyses elucidated critical mechanisms: GCS serves as a central hub mediating indirect pathways from ADL, ASPECTS, and mRS to language outcomes, while infarct volume shows a pronounced sex-specific pattern (weight = −0.31). These findings validate multimodal integration for superior prediction and reveal interconnected risk architectures overlooked by conventional models, paving the way for precision interventions targeting consciousness enhancement and sex-stratified rehabilitation strategies.

## Data Availability

The raw data supporting the conclusions of this article will be made available by the authors, without undue reservation.

## Footnotes

1
https://github.com/LeiGuo0812/quickNet
