# Supplementary material for: Sex-specific infarct volume associations and early prediction of language impairment progression following stroke surgery: a network approach
Source: Front Neurol. 2025 Dec 3;16:1728433. doi: 10.3389/fneur.2025.1728433 (PMC12708294; doi:10.3389/fneur.2025.1728433)
Supplement: Supplementary file 1 [file Table_1.docx]

**Supplement Materials**

**Content**

[**Supplement Materials** 1](#_Toc214756838)

[**Table S1.** STROBE Statement—checklist of items that should be included in reports of observational studies 2](#_Toc214756839)

[**Table S2.** Table of comparison of brain region damage among patients with different degrees of deterioration in language impairment. 6](#_Toc214756840)

[**Table S3.** Table of abbreviations and full names of predictive factors, related to Figure 2 and Figure3. 7](#_Toc214756841)

[**Figure S1.** Bootstrapped confidence intervals of estimated edge weights for the GLASSO network, related to Figure 2a. 8](#_Toc214756842)

[**Figure S2**. Bootstrapped difference test between edge weights that were non-zero in the estimated network, related to Figure 2a. 9](#_Toc214756843)

[**Figure S3.** Bootstrapped difference test between node strength of the network, related to Figure 2a. 10](#_Toc214756844)

[**Figure S4.** Stability of strength centrality index, related to Figure 2a. 11](#_Toc214756845)

[**Figure S5.** Histogram of the Resiguals of Each Symptom, related to Figure 2c. 12](#_Toc214756846)

[**Figure S6.** The Log-Likelihood Loss for the training dataset, related to Figure 2c. 13](#_Toc214756847)

[**Figure S7.** Bootstrapped confidence intervals of estimated edge weights for the NOA network, related to Figure 3a. 14](#_Toc214756848)

[**Figure S8**. Bootstrapped difference test between edge weights that were non-zero in the estimated NOA network, related to Figure 3a. 15](#_Toc214756849)

[**Figure S9.** Bootstrapped difference test between node strength of the NOA network, related to Figure 3a. 16](#_Toc214756850)

[**Figure S10.** Stability of strength centrality index, related to Figure 3a. 17](#_Toc214756851)

[**Supplementary Methods1.** Lesion Segmentation Validation 18](#_Toc214756852)

[**Supplementary Results1.** Supplementary detailed values for the table 19](#_Toc214756853)

**Table S1.** STROBE Statement—checklist of items that should be included in reports of observational studies

|  | Item No | Recommendation | Page  No |
| --- | --- | --- | --- |
| **Title and abstract** | 1 | (*a*) Indicate the study’s design with a commonly used term in the title or the abstract | 1 |
|  |  | (*b*) Provide in the abstract an informative and balanced summary of what was done and what was found | 2 |
| Introduction | | | |
| Background/rationale | 2 | Explain the scientific background and rationale for the investigation being reported | 3 |
| Objectives | 3 | State specific objectives, including any prespecified hypotheses | 3-4 |
| Methods | | | |
| Study design | 4 | Present key elements of study design early in the paper | 5 |
| Setting | 5 | Describe the setting, locations, and relevant dates, including periods of recruitment, exposure, follow-up, and data collection | 5 |
| Participants | 6 | (*a*) *Cohort study*—Give the eligibility criteria, and the sources and methods of selection of participants. Describe methods of follow-up  *Case-control study*—Give the eligibility criteria, and the sources and methods of case ascertainment and control selection. Give the rationale for the choice of cases and controls  *Cross-sectional study*—Give the eligibility criteria, and the sources and methods of selection of participants | 5 |
|  |  | (*b*) *Cohort study*—For matched studies, give matching criteria and number of exposed and unexposed  *Case-control study*—For matched studies, give matching criteria and the number of controls per case | 5 |
| Variables | 7 | Clearly define all outcomes, exposures, predictors, potential confounders, and effect modifiers. Give diagnostic criteria, if applicable | 5-6 |
| Data sources/ measurement | 8* | For each variable of interest, give sources of data and details of methods of assessment (measurement). Describe comparability of assessment methods if there is more than one group | 5-6 |
| Bias | 9 | Describe any efforts to address potential sources of bias | 5-7 |
| Study size | 10 | Explain how the study size was arrived at | 5 |
| Quantitative variables | 11 | Explain how quantitative variables were handled in the analyses. If applicable, describe which groupings were chosen and why | 5-7 |
| Statistical methods | 12 | (*a*) Describe all statistical methods, including those used to control for confounding | 6-7 |
|  |  | (*b*) Describe any methods used to examine subgroups and interactions | 5-8 |
|  |  | (*c*) Explain how missing data were addressed | 6 |
|  |  | (*d*) *Cohort study*—If applicable, explain how loss to follow-up was addressed  *Case-control study*—If applicable, explain how matching of cases and controls was addressed  *Cross-sectional study*—If applicable, describe analytical methods taking account of sampling strategy | 5 |
|  |  | (*e*) Describe any sensitivity analyses | 6-8 |

Continued on next page

| Results | | | |
| --- | --- | --- | --- |
| Participants | 13* | (a) Report numbers of individuals at each stage of study—eg numbers potentially eligible, examined for eligibility, confirmed eligible, included in the study, completing follow-up, and analysed | 8-12 |
|  |  | (b) Give reasons for non-participation at each stage | 8-12 |
|  |  | (c) Consider use of a flow diagram | Not Applicable |
| Descriptive data | 14* | (a) Give characteristics of study participants (eg demographic, clinical, social) and information on exposures and potential confounders | 8-12 |
|  |  | (b) Indicate number of participants with missing data for each variable of interest | 5, 8-12 |
|  |  | (c) *Cohort study*—Summarise follow-up time (eg, average and total amount) | 5 |
| Outcome data | 15* | *Cohort study*—Report numbers of outcome events or summary measures over time | 8-12 |
|  |  | *Case-control study—*Report numbers in each exposure category, or summary measures of exposure |  |
|  |  | *Cross-sectional study—*Report numbers of outcome events or summary measures |  |
| Main results | 16 | (*a*) Give unadjusted estimates and, if applicable, confounder-adjusted estimates and their precision (eg, 95% confidence interval). Make clear which confounders were adjusted for and why they were included | 8 |
|  |  | (*b*) Report category boundaries when continuous variables were categorized | 8 |
|  |  | (*c*) If relevant, consider translating estimates of relative risk into absolute risk for a meaningful time period | Not Applicable |
| Other analyses | 17 | Report other analyses done—eg analyses of subgroups and interactions, and sensitivity analyses | 8-14 |
| Discussion | | | |
| Key results | 18 | Summarise key results with reference to study objectives | 15 |
| Limitations | 19 | Discuss limitations of the study, taking into account sources of potential bias or imprecision. Discuss both direction and magnitude of any potential bias | 15-16 |
| Interpretation | 20 | Give a cautious overall interpretation of results considering objectives, limitations, multiplicity of analyses, results from similar studies, and other relevant evidence | 15-16 |
| Generalisability | 21 | Discuss the generalisability (external validity) of the study results | 15-16 |
|  | | | |
| Funding | 22 | Give the source of funding and the role of the funders for the present study and, if applicable, for the original study on which the present article is based | 17 |

*Give information separately for cases and controls in case-control studies and, if applicable, for exposed and unexposed groups in cohort and cross-sectional studies.

**Note:** An Explanation and Elaboration article discusses each checklist item and gives methodological background and published examples of transparent reporting. The STROBE checklist is best used in conjunction with this article (freely available on the Web sites of PLoS Medicine at http://www.plosmedicine.org/, Annals of Internal Medicine at http://www.annals.org/, and Epidemiology at http://www.epidem.com/). Information on the STROBE Initiative is available at www.strobe-statement.org.

**Table S2.** Table of comparison of brain region damage among patients with different degrees of deterioration in language impairment.

| Category | Postoperative Improvement (n=45) | Postoperative No Improvement (n=119) | t/χ2 value | p value | Adjusted p value | Effect size |
| --- | --- | --- | --- | --- | --- | --- |
| **Admission** |  |  |  |  |  |  |
| The Affected Hemisphere (left/%) | 14/31.1% | 52/43.7% | 1.66^a^ | 0.198 | 0.494 | 0.08^b^ |
| Cerebellar Damage (Yes/%) | 14/31.1% | 31/26.1% | 0.20^a^ | 0.651 | 0.820 | 0.00^b^ |
| Language Area Damage (Yes/%) | 3/6.7% | 7/5.9% |  | 1.000 | 1.000 | 0.00^b^ |
| Broca's area Damage (Yes/%) | 1/2.2% | 1/0.8% |  | 0.475 | 0.672 | 0.00^b^ |
| Wernicke 's area Damage (Yes/%) | 2/4.4% | 6/5.0% |  | 1.000 | 1.000 | 0.00^b^ |

Note: ^a^ Chi-square test. Fisher's exact test was applied to analyze the categorical variables of Language Area Damage, Broca's Area Damage, and Wernicke's Area Damage, as the assumption for the Chi-square test was violated (i.e., expected counts in these variables were < 5). Statistically significant differences are marked with a p-value less than 0.05. Effect sizes were assessed using ^b^ Cramer's V for chi-square and fisher's exact test. Some values appear as 0 due to their very small magnitude; however, for statistical measures that include directionality and require verification, please refer to Supplementary Results1.

**Table S3.** Table of abbreviations and full names of predictive factors, related to Figure 2 and Figure3.

| Abbreviation | Full Name of Variable |
| --- | --- |
| PQ3 | ADL score at admission |
| PQ5 | GCS score at admission |
| PSQ3 | ASPECTS score after surgery |
| PSQ6 | mRS score at discharge |
| BIOC4 | C-reactive protein 0-10 mg/l |
| BIOC19 | Glycated hemoglobin 4-6% |
| VOL | Infarct volume |
| CE1 | The Affected Hemisphere |
| LANG | Language deterioration score |

Note: ASPECT, Alberta Stroke Program Early CT Score; mRS, Modified Rankin Scale; ADL, Activities of Daily Living; GCS, Glasgow Coma Scale.

**Figure S1.** Bootstrapped confidence intervals of estimated edge weights for the GLASSO network, related to Figure 2a.


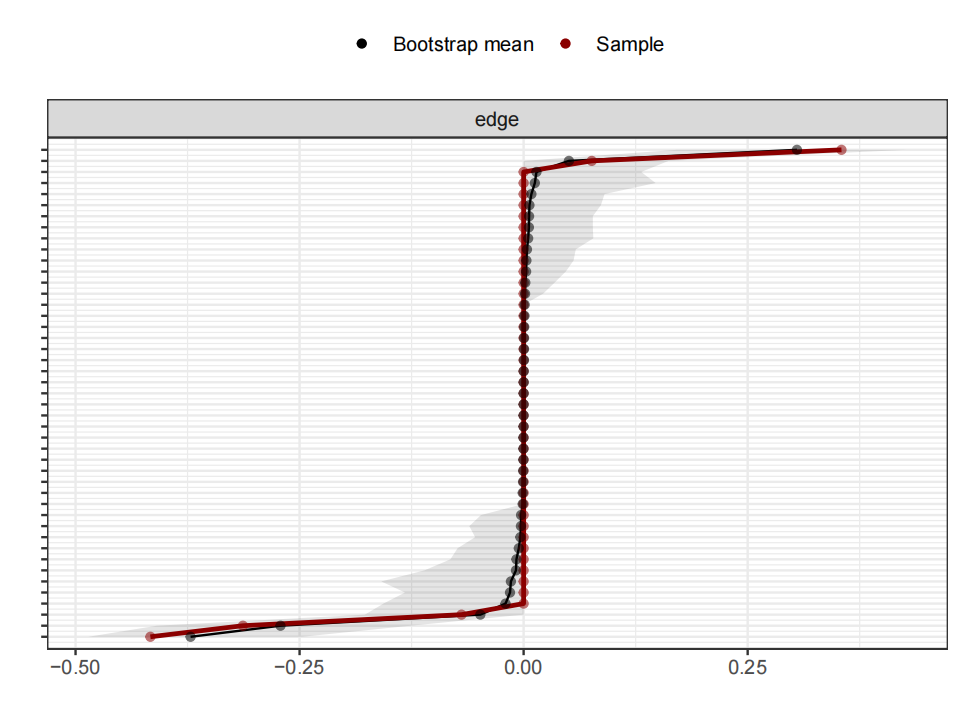


The red line indicates the sample values and the grey area the bootstrapped CIs.

**Figure S2**. Bootstrapped difference test between edge weights that were non-zero in the estimated network, related to Figure 2a.


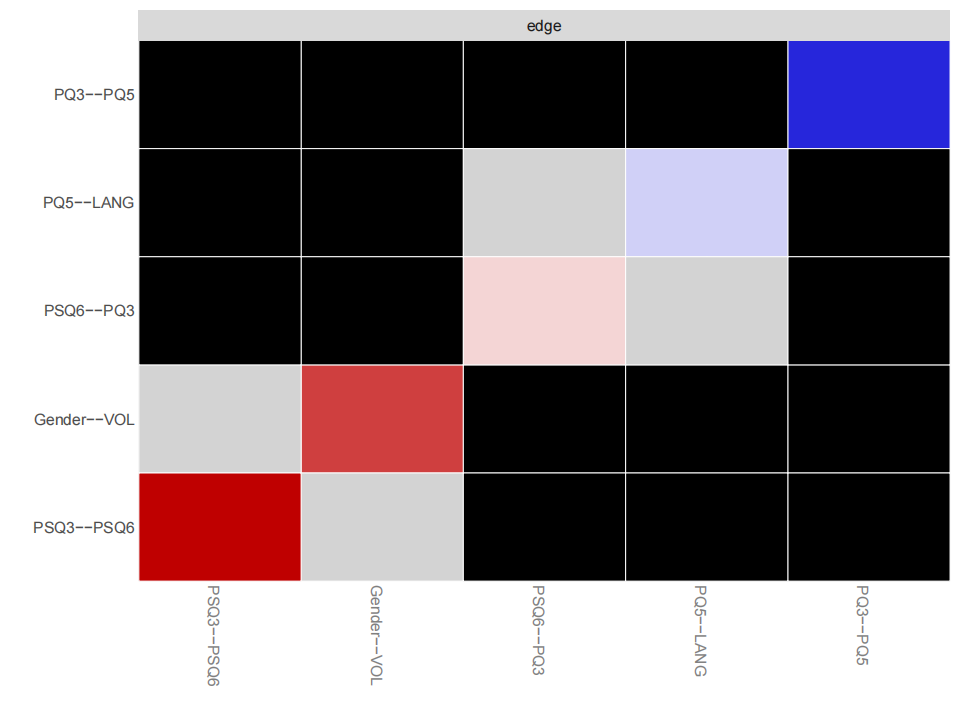
Grey boxes indicate edges that do not differ significantly from one another and black boxes represent edges that differ significantly from one another.

**Figure S3.** Bootstrapped difference test between node strength of the network, related to Figure 2a.


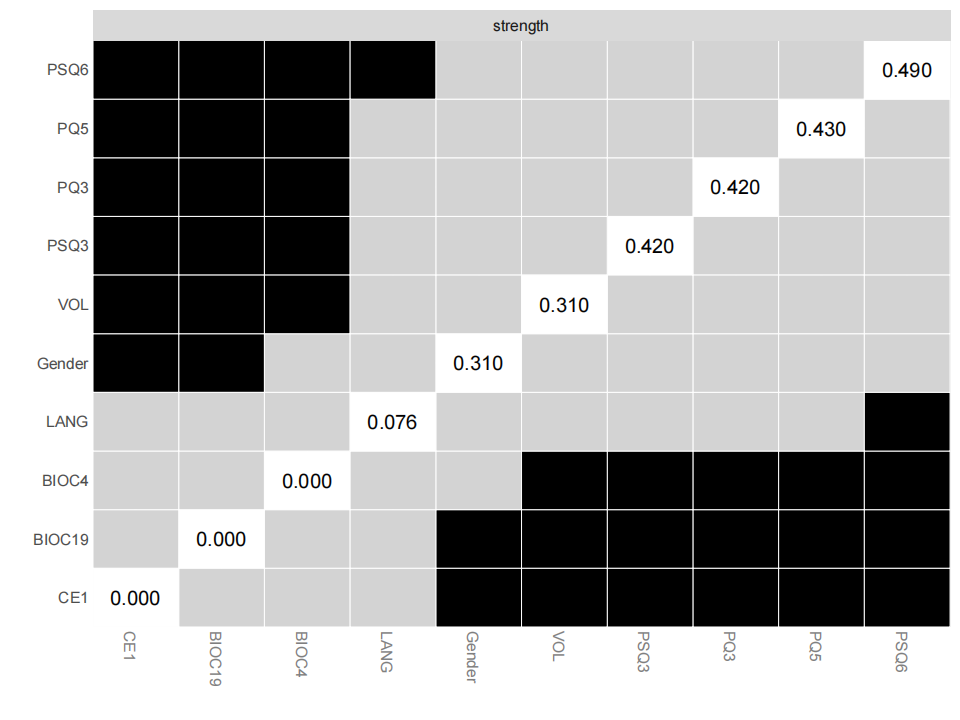


Grey boxes indicate nodes that do not differ significantly from one another and black boxes represent nodes that differ significantly from one another. The number in the white boxes (i.e., diagonal line) represent the value of node expected influences.

**Figure S4.** Stability of strength centrality index, related to Figure 2a.


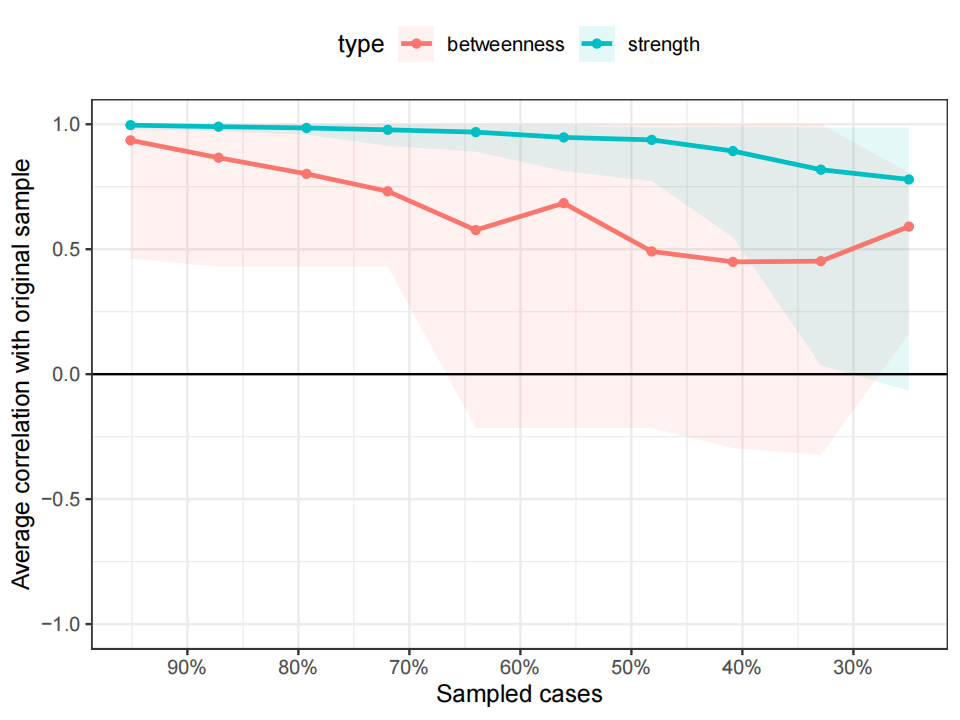


The red, green and blue lines represents the average correlation between betweenness, closeness and strength in the full sample and subsample.

**Figure S5.** Histogram of the Resiguals of Each Symptom, related to Figure 2c.


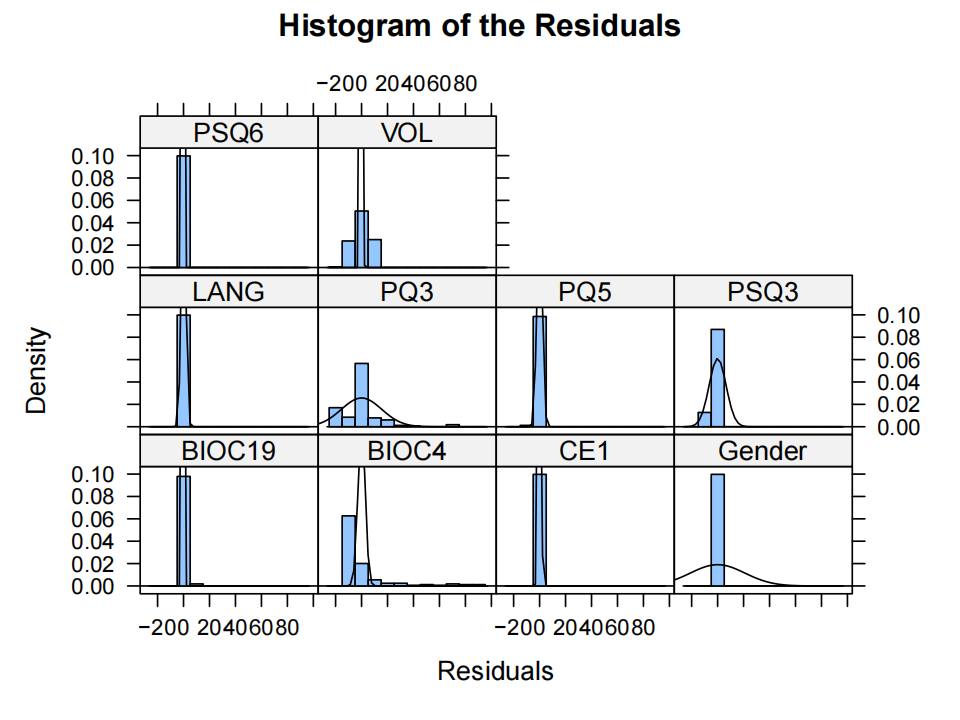


Residual distribution of subjects in each symptom.

Note: Language predictive factors include PSQ3: ASPECTS postoperative score; PSQ6: MRS discharge score; BIOC4: C-reactive protein (0–10 mg/L); BIOC19: glycosylated hemoglobin (4–6%); PQ3: ADL admission score; PQ5: GCS admission score; CE1, the affected hemisphere; VOL: cerebral infarction volume; and LANG: degree of language deterioration.

**Figure S6.** The Log-Likelihood Loss for the training dataset, related to Figure 2c.


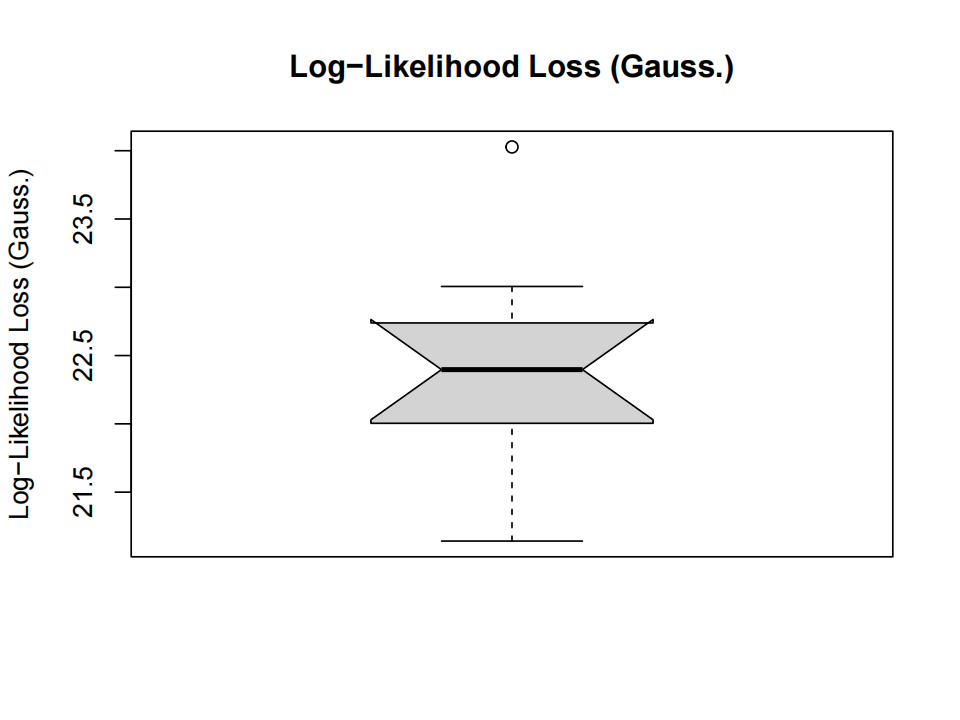


Log-Likelihood Loss also known as negative entropy or negentropy, it is the negated expected log-likelihood of the test set for the Bayesian network fitted from the training set. Mean_loss = 22.38, SD_loss=0.81.

**Figure S7.** Bootstrapped confidence intervals of estimated edge weights for the NOA network, related to Figure 3a.


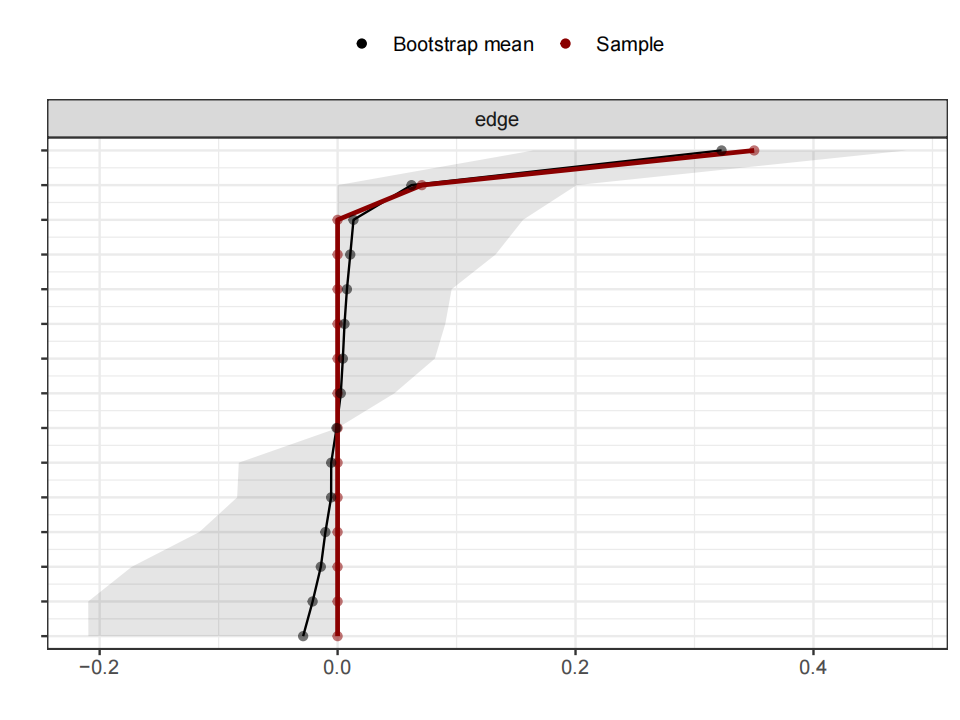


The red line indicates the sample values and the grey area the bootstrapped CIs.

**Figure S8**. Bootstrapped difference test between edge weights that were non-zero in the estimated NOA network, related to Figure 3a.


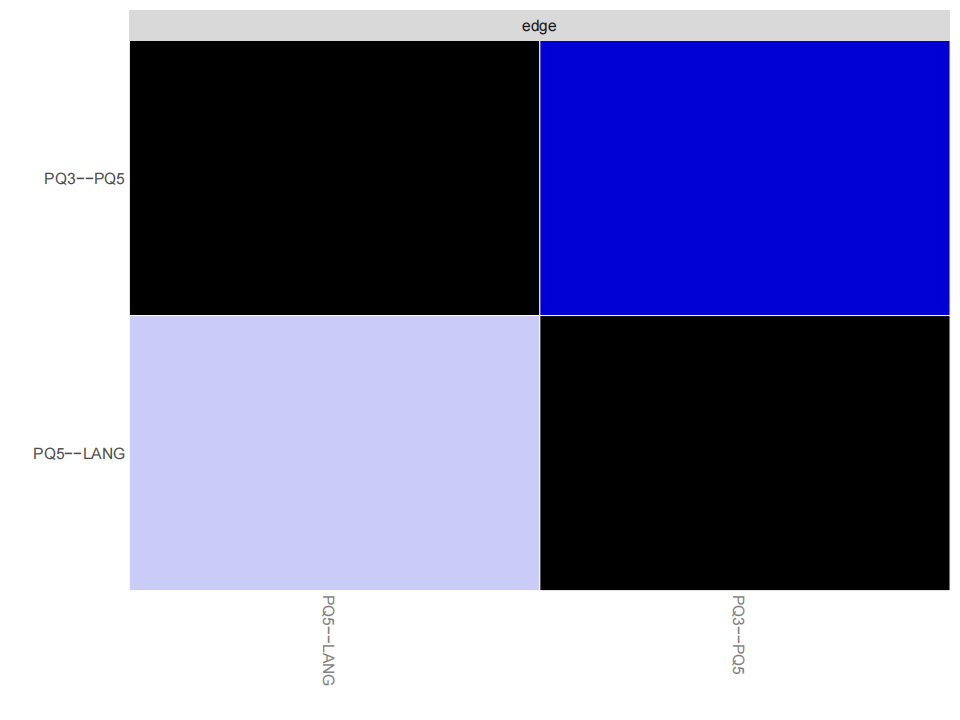
Grey boxes indicate edges that do not differ significantly from one another and black boxes represent edges that differ significantly from one another.

**Figure S9.** Bootstrapped difference test between node strength of the NOA network, related to Figure 3a.


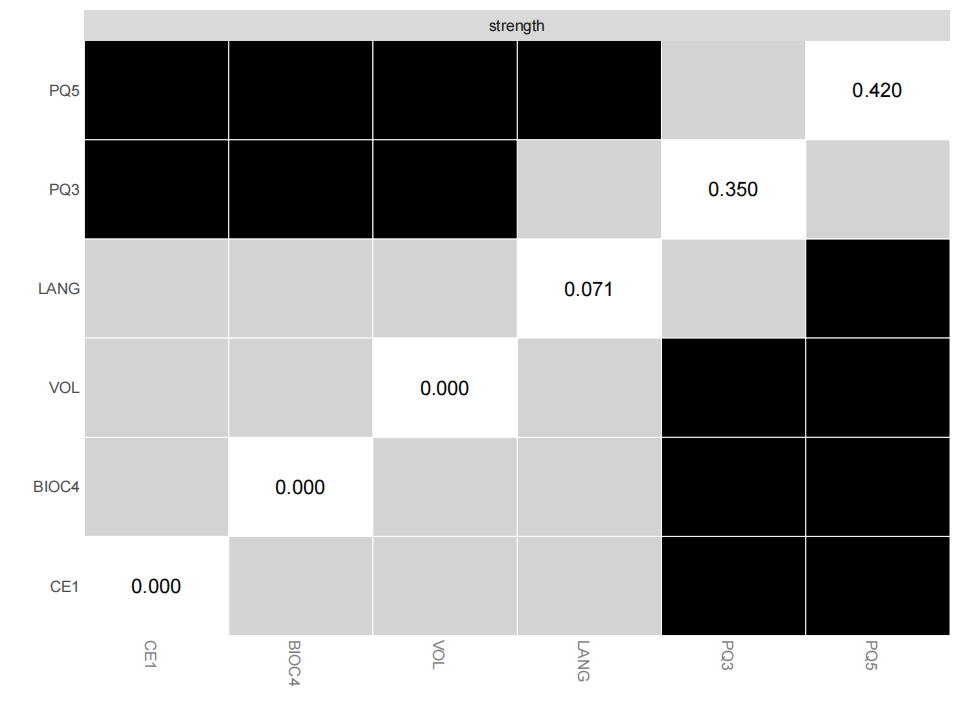


Grey boxes indicate nodes that do not differ significantly from one another and black boxes represent nodes that differ significantly from one another. The number in the white boxes (i.e., diagonal line) represent the value of node expected influences.

**Figure S10.** Stability of strength centrality index, related to Figure 3a.


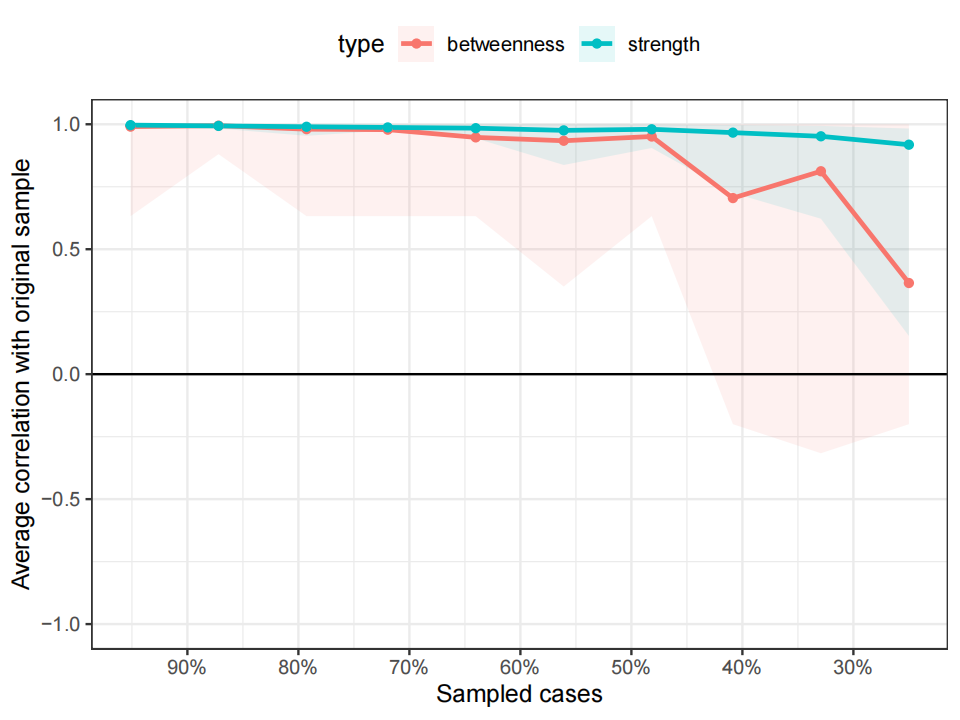


The red, green and blue lines represent the average correlation between betweenness, closeness and strength in the full sample and subsample.

**Supplementary Methods1.** Lesion Segmentation Validation

To enhance methodological transparency and reproducibility, we have added a new Supplementary Methods section describing the semi-automated lesion quantification pipeline used in this study. Cerebral infarct volumes were derived from admission non-contrast CT (NCCT) scans using a hemispheric asymmetry framework implemented in the FMRIB Software Library (FSL, v6.0). Each scan was first reoriented to the standard neurological convention (fslreorient2std), skull-stripped with a CT-optimized fractional intensity threshold (BET, f = 0.10), and resampled to 1.0 mm isotropic resolution to standardize voxel dimensions. Image intensities were then normalized within a Hounsfield Unit (HU) window of –20 to 80 to enhance the contrast of ischemic hypoattenuation relative to normal brain tissue.

To capture lesion-related hemispheric asymmetry, a left–right flipped version of the normalized brain was generated and rigidly registered back to the original image (6 degrees of freedom, normalized mutual information cost function). An absolute voxel-wise difference map between the original and registered flipped image was computed, yielding an asymmetry map in which ischemic lesions appear as regions of high intensity. This map was thresholded at the 98th percentile of its intensity distribution to obtain a binary lesion candidate mask, and small isolated clusters with a volume < 500 mm³ (500 voxels at 1 mm³) were removed using a cluster-extent filter. The resulting lesion mask was then rigidly aligned to the MNI152_T1_1mm standard space (FSL standard template) to allow spatially standardized quantification across subjects.

From the MNI-aligned lesion masks, we extracted lesion volume (mm³ and mL), lesion centroid coordinates (x, y, z), and several derived regional indices. Hemispheric laterality was defined based on the MNI x-coordinate of the lesion centroid (left hemisphere = 1 if x < 0, right hemisphere = 0 if x ≥ 0). Cerebellar involvement was flagged when the centroid’s z-coordinate was below –30 in MNI space, which approximately corresponds to the infratentorial cerebellar region. To characterize language-related involvement in an anatomically interpretable but automated manner, we further computed proximity to two canonical left-hemisphere language-related regions using simple MNI bounding boxes: an approximate “Broca-like” region (x ∈ [–60, –20], y ∈ [10, 70], z ∈ [–10, 40]) and an approximate “Wernicke-like” region (x ∈ [–60, –20], y ∈ [–60, –10], z ∈ [–10, 20]). A binary indicator of “any language-related involvement” was set to 1 if the centroid fell within either of these regions.

All lesion masks and derived spatial summaries underwent visual quality control in FSLeyes by experienced raters to verify anatomical plausibility and to ensure that major ischemic territories were appropriately captured. The overall approach is conceptually consistent with prior work that exploits inter-hemispheric mirror differences for stroke lesion mapping and segmentation (e.g., Bao et al., 2022, IEEE J Biomed Health Inform 26(4):1628–1639), while our implementation is specifically tailored to admission NCCT and the constraints of the current cohort.

**Supplementary Results1.** Supplementary detailed values for the table

Some values appear as 0 due to their very small magnitude; however, the directionally informed statistical values are presented in detail as follows.

**Table1**

**Wernicke’s area Damage (Yes/%) Effect size**: 1.61486619355413E-16

**Neutrophil (10^9) Effect size**: -0.000679675935045226

**Neutrophil (10^9) t value**: -0.00492964273344702

**Triglycerides (mmol/L) Effect size**: -0.00116073075026371

**Table2**

**Language Area Damage (Yes/%) Effect size**: 3.07016070570611E-16

**Broca’s area Damage (Yes/%) Effect size**: 2.44238931666757E-17

**Wernicke’s area Damage (Yes/%) Effect size**: 4.47734853340436E-16

**D-Dimer (ng/mL) Effect size**: 0.00209777595920248
